# Interactions of polymyxin B with lipopolysaccharide-containing membranes

**DOI:** 10.1039/d1fd00036e

**Published:** 2021-07-26

**Authors:** Alice Goode, Vivien Yeh, Boyan B. Bonev

**Affiliations:** School of Life Sciences, University of Nottingham, Queen’s Medical Centre Nottingham NG7 2UH UK boyan.bonev@nottingham.ac.uk

## Abstract

Bacterial resistance to antibiotics constantly remodels the battlefront between infections and antibiotic therapy. Polymyxin B, a cationic peptide with an anti-Gram-negative spectrum of activity is re-entering use as a last resort measure and as an adjuvant. We use fluorescence dequenching to investigate the role of the rough chemotype bacterial lipopolysaccharide from *E. coli* BL21 as a molecular facilitator of membrane disruption by LPS. The minimal polymyxin B/lipid ratio required for leakage onset increased from 5.9 × 10^−4^ to 1.9 × 10^−7^ in the presence of rLPS. We confirm polymyxin B activity against *E. coli* BL21 by the agar diffusion method and determined a MIC of 291 μg ml^−1^. Changes in lipid membrane stability and dynamics in response to polymyxin and the role of LPS are investigated by ^31^P NMR and high resolution ^31^P MAS NMR relaxation is used to monitor selective molecular interactions between polymyxin B and rLPS within bilayer lipid membranes. We observe a strong facilitating effect from rLPS on the membrane lytic properties of polymyxin B and a specific, pyrophosphate-mediated process of molecular recognition of LPS by polymyxin B.

## Abstract 2

Polymyxin B uses bacterial LPS as docking receptor to cross the outer membrane.

## Graphical abstract

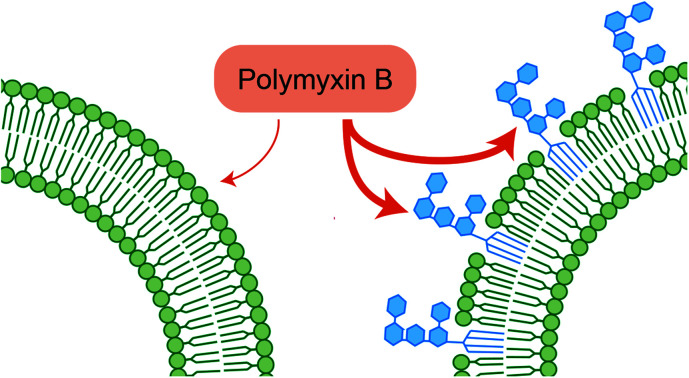

## Introduction

Bacterial infections remain the primary cause of morbidity and mortality in humans despite the success of antibiotic chemotherapy. Bacteria have natural defences against xenobiotics but also the ability to adapt as a population under sustained antibiotic pressure that selects favourable phenotypes. In addition, such adaptations can be transferred within and between bacterial populations, which leads to a growing prevalence of bacterial phenotypes that do not respond well or are resistant to antibiotics. Particularly challenging are Gram-negative bacteria, which have an outer membrane (OM) that hinders antibiotic accessibility to target sites and reduces bacterial susceptibility to antibiotics. One approach to the management of such infections is the use of adjuvants in combination with conventional antibiotics.

The definitive feature of Gram-negative bacteria is the presence of an outer membrane that envelopes the entire bacterial cell and forms a periplasmic compartment containing bacterial peptidoglycan and a number of specialised enzymes, often with roles in the post-translational modification of proteins. The bacterial OM is an asymmetric bilayer with an inner leaflet made largely of phospholipids common to the inner membrane (IM), while the outer leaflet of the OM is almost entirely made of lipopolysaccharide (LPS). The OM also contains a significant fraction of membrane proteins with a unique, beta-barrel fold.

**Fig. 1:**
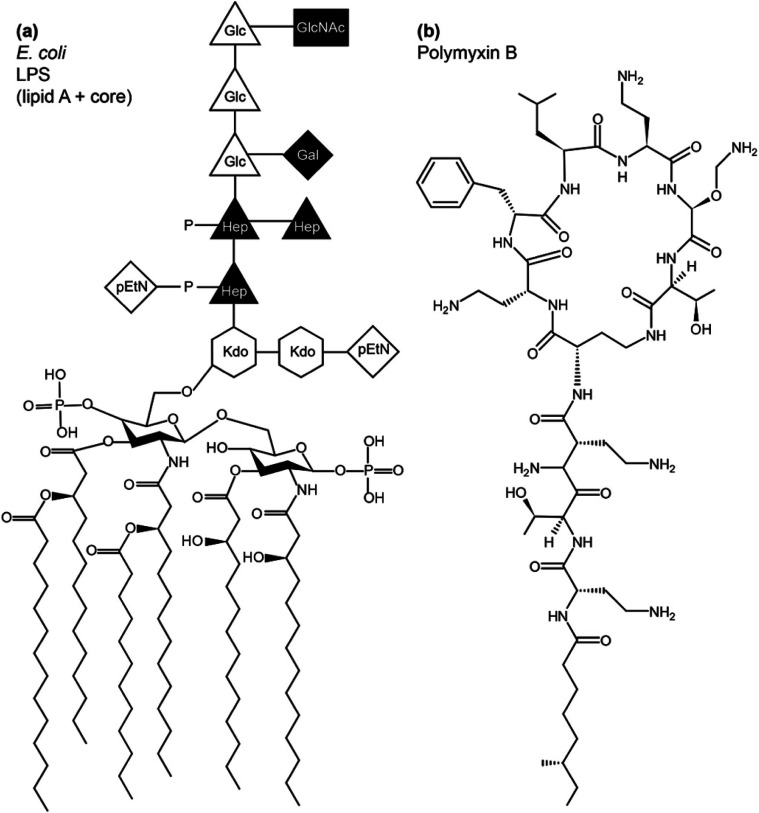
Schematic structure of LPS from *E. coli* (a) and of polymyxin B (b).

LPS lines the bacterial exterior where it performs a number of important functions, including cell recognition and xenobiotic defence. While the molecular structure of LPS has unique features that identify the bacterial species, its overall architecture commonly contains three parts – lipid A, the core oligosaccharide and O-antigen (Fig. 1). Lipid A has a largely conserved structure, which comprises a N-acetylated disaccharide, commonly derivatised at positions 2,3,2′ and 3′ with six or seven 14-carbon saturated chains and often phosphorylated or pyrophosphorylated in positions 1 and 4′. Also conserved, the polysaccharide core attached at 6′ on lipid A contains unique 3-deoxy-d-*manno*-oct-2-ulosonic acid (Kdo) and heptose (Hep) monosaccharides that can be phosphorylated, pyrophosphorylated or derivatised with phosphoethanolamine (pEtN). A variable length, species-specific O-antigen, consisting of up to 40–50 saccharide repeats, extends beyond the core and serves as the characteristic recognition motif that identifies the bacterial species. LPS that has all three components is of the smooth chemotype (sLPS), while LPS lacking the O-antigen is of the rough chemotype (rLPS). Commonly studied *Escherichia coli* strains BL21 and K12 have truncated rLPS (Fig. 1). Phosphorylation and pyrophosphorylation in LPS are essential for establishing and maintaining OM stability *via* divalent cation-mediated (chiefly Ca^2+^ or Mg^2+^) LPS/LPS interactions. Using solid state NMR, we have shown recently that such phosphorylation and pyrophosphorylation is extensive in the core region but sub-stoichiometric within lipid A, revealing the pivotal role of the core in OM stability.^1^

The conserved structure of LPS has driven co-evolution of antibacterial proteins,^2^ peptides^3^ and other xenobiotics that rely on molecular LPS recognition to disrupt bacterial OMs and exercise antibacterial functions. Polymyxins and colistins are a family of cyclic polycationic peptides derived from progenitors naturally produced by *Bacillus polymyxa*.^4^ They were in clinical use until the 1980s, when their use declined due to relatively high nephrotoxicity.^5^ Due to the rise in antibiotic resistance, despite their nephrotoxicity, polymyxins find use as last defence drugs against carbapenem-resistant Gram-negative infections^6^ and can be used as adjuvants in antibiotic cocktails.^7^

Polymyxins disrupt both the outer and inner bacterial membranes.^8^ Crossing the OM is sensitive to variation in the LPS chemotype, suggesting an LPS-dependent receptor-specific mechanism, while crossing the IM is sensitive to lipid composition. Besides the disruption of both membranes, cellular targets have been suggested to include DNA and ribosomes where polymyxin induces DNA clotting and ribosomal condensation.^9,10^ The mechanism of antimicrobial action and bacterial adaptations and resistance have been reviewed recently.^11^

Modifications of LPS, specifically the pEtN-ation of lipid A, reduce susceptibility to polymyxins through the reduction of the LPS negative charge (for a review see ref. 12). Polymyxin resistant strains of *P. aeruginosa* have been shown to produce less phospholipid,^13^ most likely through a pressure selection rather than as an adaptive response in protecting the bacterial IM. A study of polymyxin mediated glucose release from lipid vesicles has shown reduced release from phosphatidylcholine and methyl phosphatidyl ethanolamine compared to phosphatidyl ethanolamine membranes.^14^

In this report, we investigate the role of rLPS as a molecular receptor for polymyxin in lipid membranes using fluorescence dequenching and we monitor its antimicrobial activity. We investigate specific molecular interactions between polymyxin and LPS using longitudinal relaxation ^31^P magic angle spinning (MAS) NMR. Relative changes in fast molecular dynamics are compared to the membrane phospholipid. We monitor membrane stability and collective dynamics by following changes in the ^31^P chemical shift anisotropy (CSA) using wideline ^31^P solid state NMR.

## Experimental

Phospholipids, 1,2-dioleoyl-*sn-glycero*-3-phosphocholine (DOPC) and 1,2-dimyristoyl-*sn-glycero*-3-phosphocholine (DMPC) (Avanti Polar Lipids Inc. Alabaster, Alabama, USA) were used as purchased at >98% purity.

### Phenol extraction of rLPS

Rough chemotype lipopolysaccharide (rLPS) was purified from *E. coli* (BL21) as previously described.^15,16^ Briefly, 3 g of the *E. coli* (BL21) bacterial pellet was resuspended in deionised water and heated to 68 °C. To this, 90% phenol (w/v) was added dropwise and stirred at 68 °C, cooled on ice for 15 min and centrifuged at 1000*g* for 45 min at 10 °C. The aqueous phase was collected and a further 10 ml of deionised water at 68 °C was added to the remaining sample, which was cooled and centrifuged and the aqueous phase was collected and dialysed to remove any remaining phenol, then freeze dried. The crude product was resuspended in 0.1 M Tris pH 7.0 and 175 mM NaCl, 0.05% w/v sodium azide at 10 mg ml^−1^ and underwent a further purification process, as described previously.^16^ Briefly, the sample was treated with DNase and RNase (both 50 μg per 10 mg of crude LPS) at 37 °C for 30 min. This was followed by treatment with proteinase K (50 μg per 10 mg of crude LPS) at 55 °C for 3 h and then at room temperature for 16 h with fresh proteinase K added. The proteinase K treatments were repeated and LPS was precipitated with 4 volumes of methanol at 18 °C for 2 h. The precipitate was recovered by centrifugation at 6000*g* for 15 min at 4 °C and freeze-dried.

### LPS-containing membranes

LPS was suspended in distilled water and incubated at 56 °C for 15 min, vortexed for 2 min and then cooled to 4 °C. This was repeated thrice. Samples were incubated with either DOPC (Avanti) (1 : 1 w/w ratio of DOPC : LPS) or DMPC (Avanti) (2 : 1 ratio of DMPC : LPS w/w) prepared as small unilamellar vesicles by sonication, as previously described.^16^ The sample was then freeze dried.

### Polymyxin B preparation

20 mg of polymyxin B (Fisher bioreagents) was resuspended in 1 ml HPLC water and then centrifuged for 10 min at 3000*g* to remove precipitates. This was then loaded onto a C18 RP-HPLC column. The elution of polymyxin B was with a linear gradient from 3.5% to 70% acetonitrile with 0.1% v/v TFA over 30 min, measured using a UV-vis detector at 220 nm. The acetonitrile was then removed and a small amount was sent to ESI MS (Bruker MicroTOF) to confirm the presence of polymyxin B. Polymyxin B was resuspended at 50 mg ml^−1^ in HPLC water and then lyophilised LPS in DMPC was either hydrated in HPLC water or polymyxin B in HPLC water (50 mg ml^−1^). Polymyxin B was added at a 1 : 1 molar ratio of LPS : polymyxin B. When the samples were completely hydrated and mixed with a glass stirring rod, they were freeze–thawed 5 times and loaded into 4 mm MAS NMR rotors.

### Solid state NMR

Solid state NMR experiments were carried out on a Varian 400 MHz VNMRS widebore spectrometer equipped with a 4 mm T4 MAS NMR probe. The temperature was regulated with balanced heated/vortex tube-cooled gas flow.^17^ The phosphorus-31 MAS NMR were referenced externally to H_3_PO_4_ at 0 ppm at a frequency of 161.82 MHz.

The results of the phosphorous-31 static wideline NMR experiments were recorded at 28 °C, above the transition temperature of DMPC, using a Hahn echo sequence with 100 kHz π/2 and π pulses separated by 12 μs intervals and preacquisition delays. Spectra were recorded with 20 ms acquisition time with a recycle delay of 5 s, under SPINAL-64 heteronuclear decoupling.^18^

The results of the high resolution ^31^P experiments were acquired at 5 kHz MAS frequency at either 4 °C or 28 °C. Inversion recovery was used to investigate the ^31^P longitudinal relaxation, with delay times varying between 10 ms and 1.5 s between initial π pulses and π/2 reading pulse. Spectra were recorded with 50 ms acquisition time under SPINAL64 decoupling^18^ with a recycle delay of 9 s to exceed five-fold ^31^P *T*_1_ values in membranes. Relaxation times *T*_1_ were obtained assuming a single exponential relaxation mechanism by fitting
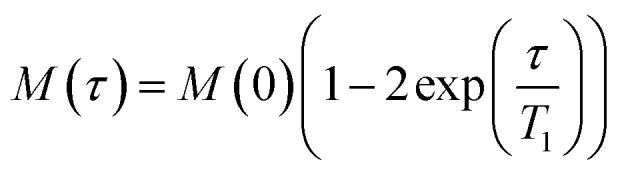


using Excel (Microsoft). All spectra were processed and analysed using ACDLabs 2015.

### Dye release fluorescence studies

Carboxyfluorescein (CF) fluorescence dequenching studies were performed as previously described.^15^ Briefly, polymyxin B was suspended in 100 mM NaCl and 10 mM Hepes (pH 7.4) and equilibrated overnight at 4 °C. DOPC films or DOPC : rLPS (1 : 1 w/w) were hydrated in 1 ml 5(6)-carboxyfluorescein (Acros organics) buffer (50 mM CF, 50 mM NaCl, 10 mM Hepes pH 7.4) for 1 h. The solution then underwent 5 cycles of freeze–thawing until the lipid films were fully suspended. The resulting suspension was extruded 11 times through a 100 nm polycarbonate filter with an Avanti extruder (Avanti Polar Lipids). CF-loaded vesicles were separated from non-encapsulated CF using a PD-10 column (GE Healthcare) equilibrated with 100 mM NaCl in 10 mM Hepes, pH 7.4, and used within 24 h.

Polymyxin B-induced CF release was monitored using the fluorescence increase (excitation 490 nm, emission 515 nm, 400 V) over 300 s, at which the time intensity changes with time were within 1%. CF-loaded large unilamellar vesicles (LUVs) in buffer (100 mM NaCl, 10 mM Hepes, pH 7.4) were equilibrated to achieve steady background fluorescence. Polymyxin B was added after 60 s (with a final concentration range of 0.025–50 μg ml^−1^). After equilibrium (120 s), residual liposomes were dispersed with Triton X-100 (Fluka BioChemika). For each polymyxin B concentration, experiments were repeated in triplicate. CF leakage was expressed as a fraction of CF release upon Triton X-100 addition, normalised to background fluorescence:% leakage = (*F*_pol_ − *F*_0_)/(*F*_*T*_*x*__ − *F*_0_) × 100where *F*_0_ is the baseline fluorescence recorded before the addition of polymyxin B, *F*_pol_ is the steady state fluorescence after adding polymyxin B and *F*_*T*_*x*__ is the maximum fluorescence release after the addition of Triton X-100 to destroy any remaining vesicles.

### Antimicrobial susceptibility testing

Bacterial susceptibility to polymyxin was assessed by the agar diffusion method to determine the minimal inhibitory concentration (MIC), as described previously.^19^ Briefly, 2 × 10^7^ colony forming units (CFU) of BL21 *E. coli* inocula were spread onto solid agar Luria–Bertani (LB) plates, 3 mm holes were punched with a sterile cork borer and the wells were filled with 9 μl of varying concentrations of polymyxin B between 5 and 0.625 mg ml^−1^. Plates were incubated at 37 °C for 16 h and the polymyxin diffusion distances measured from the size of bacterial growth inhibition zones less the well diameter. Plates were individually analysed and three biological repeats of three technical plates (nine repeats per point) were taken to determine the final MIC. The MIC was determined using the webtool http://www.agardiffusion.com.

## Results & discussion

### Role of LPS in membrane disruption by polymyxin

To investigate the role LPS plays as a receptor for polymyxin, we used a dye release assay, in which CF-loaded large unilamellar vesicles (LUV) of DOPC without or with *E. coli* rLPS were incubated with increasing amounts of polymyxin B. The choice of phosphatidyl choline (PC) ensured membrane stability and lack of non-specific interactions between polymyxin B and the lipid headgroups. While in Gram-negative bacterial membranes phosphatidyl ethanolamine (PE) is present at 70–80% molar fraction, unsupported bilayers favour negative curvature and show higher instability in the presence of polymyxin than PC.^14^

**Fig. 2:**
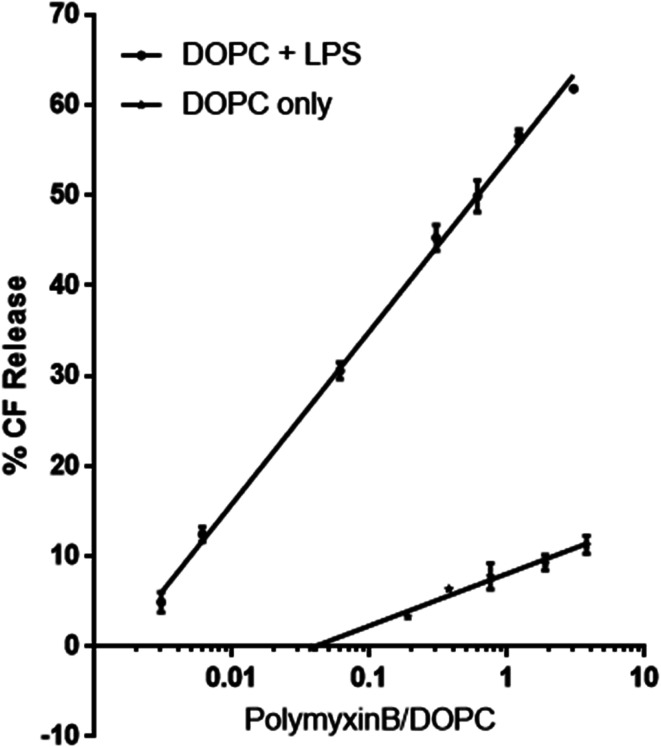
Polymyxin-dependent CF release from membranes.

CF-loaded LUVs of DOPC alone or of DOPC containing 1 : 1 w : w rLPS were incubated with polymyxin B at molar ratios of polymyxin to DOPC between 0.003 and 3 (Fig. 2). The molecular weight of rLPS was estimated at roughly 2400 ± 100 Da from the ^31^P MAS NMR phosphate (P) to pyrophosphate (PP) intensities, as previously described.^3^ To estimate the impact of LPS on membrane stability, we extrapolated the linear fits from the CF release and used the zero intercept to obtain the minimal polymyxin/DOPC ratios required for inducing lysis. Leakage onset in pure DOPC liposomes was observed at a polymyxin/DOPC ratio of 5.9 × 10^−4^, while the presence of rLPS reduced this to 1.9 × 10^−7^.

### Polymyxin binding to membranes and changes in lipid dynamics

**Fig. 3:**
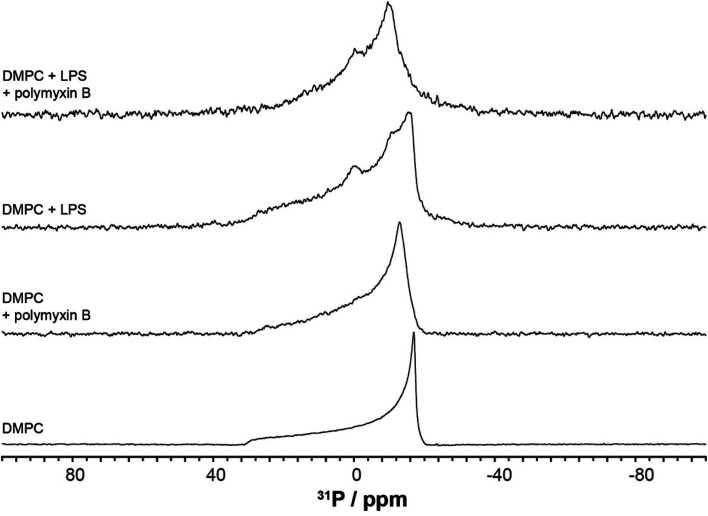
Phosphorus-31 wideline NMR spectra of the membranes, recorded at 28 °C, above the DMPC main transition temperature.

We used wideline ^31^P solid state NMR to monitor changes in the molecular organisation and slow collective dynamics in DMPC membranes that result from the addition of polymyxin, the presence of rLPS or both (Fig. 3). The ^31^P NMR wideline spectrum from hydrated DMPC in the liquid crystalline phase at 28 °C is reflective of the fast axial motions of phospholipid molecules with spherically symmetric angular orientations. The spectroscopic features are characterised by axially symmetric effective chemical shift anisotropy, which results in a characteristic powder or Pake spectral intensity distribution.^20^ The observed ^31^P effective CSA from the DMPC multilamellar vesicle (MLV) suspensions is approximately 45 ppm (Fig. 3), which is consistent with reported values.^21,22^ The addition of polymyxin at a 1 : 1 molar ratio does not disrupt the membrane structure but markedly increases the lipid disorder. The spectral features remain reflective of a powder distribution but at a much reduced effective CSA of 38.3 ppm (Fig. 3), clearly revealing the incorporation of polymyxin B into the DMPC bilayers.

The ^31^P wideline NMR spectrum from DMPC/LPS 1 : 1 w/w reveals the self-assembly of a stable bilayer membrane, reflected in the CSA-dominated powder distribution with a slightly reduced width of 43.6 ppm (Fig. 3). In addition to the DMPC-dominated symmetric CSA wideline, we observe a much more mobile environment at −7.2 ppm, reflective of the increased mobility in the lipid A region, as well as a broad isotropic resonance at 3.1 ppm. The latter arises from the LPS phosphates and pyrophosphates in the outer core.^2,3,23^ In contrast to the phospholipid phosphates, due to the high flexibility between lipid A and the LPS core, the phosphate and pyrophosphate CSA collapses completely and we observe isotropic resonances superimposed onto the DMPC wideline spectrum. Such high phosphate and pyrophosphate mobilities are also observed in the membrane embedded polyisoprenoid cell wall intermediates lipid II, lipid I and undecaprenyl mono- and pyrophosphate.^24,25^

The addition of polymyxin B at a 1 : 1 ratio to the DMPC/rLPS bilayers also preserved the powder distribution and the underlying mixed phospholipid bilayers. Akin to DMPC alone, the incorporation of polymyxin B into the mixed DMPC/rLPS membranes further increases lipid disorder compared with that in pure DMPC, which is observed as a greater reduction in the effective CSA to approximately 30 ppm.

### Selective targeting of LPS by polymyxin

**Fig. 4:**
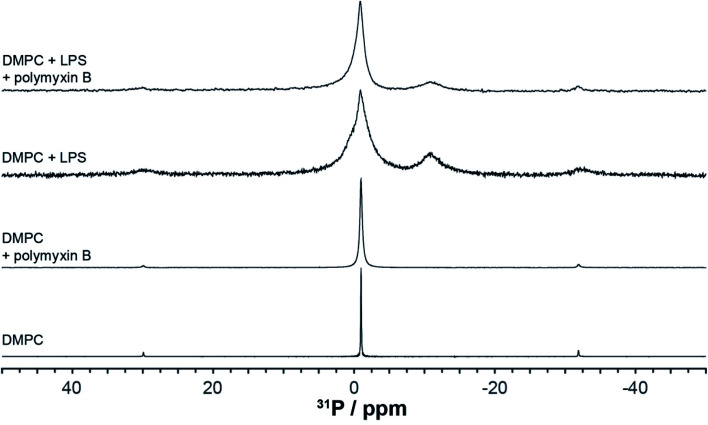
High resolution ^31^P MAS NMR spectra from DMPC or DMPC/LPS membranes without or with polymyxin, acquired at 28 °C and 5 kHz MAS.

High resolution ^31^P MAS NMR spectroscopy permits the independent and quantitative observation of individual membrane components and the selective effects of polymyxin addition. Due to pyrophosphorylation, membrane rLPS can be followed by a well resolved resonance at −11 ppm (Fig. 4). The single sharp resonance of DMPC observed at −0.97 ppm is broadened slightly upon the addition of polymyxin B. The presence of LPS in the DMPC membranes leads to a significant increase in the isotropic line width, which is countered slightly by the addition of polymyxin B (Fig. 4). Despite this line broadening, the pyrophosphate resonance remains clearly resolved from the compound intensity of DMPC and LPS monophosphates. A contribution from the 3.1 ppm LPS phosphate is seen as a shoulder on the main monophosphate resonance.

Longitudinal nuclear relaxation is a sensitive reporter of changes in fast, GHz molecular motions, such as the axial rotation in membrane lipids that leads to CSA and dipolar coupling modulation. We use inversion recovery ^31^P MAS NMR to determine the relaxation behaviour of the lipid and LPS ^31^P nuclear systems and to explore specific molecular interactions between polymyxin B and rLPS within the DMPC membranes. Phosphorus-31 within phosphates or pyrophosphates is a particularly appropriate nuclear reporter, as the pure O-linking severely reduces coupling to protons and obviates the need of decoupling during the long relaxation intervals.

**Fig. 5:**
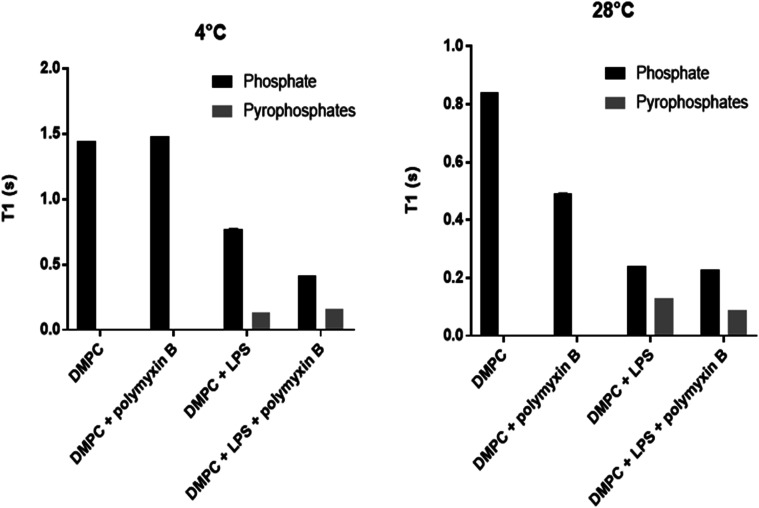
Phosphorus-31 MAS NMR longitudinal relaxation times *T*_1_ for DMPC membranes without or with LPS or polymyxin. Phosphate *T*_1_ values are shown in black and pyrophosphate *T*_1_ values from LPS are in grey.

Longitudinal ^31^P MAS NMR relaxation times *T*_1_ were determined at 28 °C and at 4 °C (Fig. 5, Table 1). At 28 °C both DMPC and rLPS are in the fast motion regime and we observe a reduction in the rLPS pyrophosphate *T*_1_ from 130 to 90 ms, while the DMPC-dominated monophosphate *T*_1_ remained almost unaffected at 240 and 230 ms, respectively. The selective reduction in the pyrophosphate relaxation time reflects reduced mobility and the formation of an LPS/polymyxin membrane complex. By contrast, the DMPC mobility remained unchanged, which is consistent with the previously reported lack of molecular interactions.^14^

**Table 1:** Phosphorus-31 MAS NMR longitudinal relaxation times *T*_1_ at 4 °C and 28 °C from DMPC membranes without or with LPS before or after polymyxin B (PMB) addition

	*T*_1_ [s] at 4 °C		*T*_1_ [s] at 28 °C	
	P	PP	P	PP
DMPC	1.44		0.84	
DMPC/PMB	1.48		0.49	
DMPC/LPS	0.77	0.13	0.24	0.13
DMPC/LPS/PMB	0.42	0.16	0.23	0.09

The relaxation time *T*_1_ from LPS pyrophosphates remained unchanged at 130 ms when the temperature was lowered from 28 °C to 4 °C, suggesting that the system is crossing a *T*_1_ minimum and enters slow motion at the lower temperature. The molecular motions are in the slow regime within DMPC in the gel phase and the reduction in the phosphate *T*_1_ upon the addition of polymyxin B reflects the increase in membrane disorder, consistent with the wideline and high resolution ^31^P MAS observations. The LPS pyrophosphate relaxation time increased at 4 °C from 130 to 160 ms, which is reflective of motional restrictions in a slow motion system and of molecular complex formation, specifically between membrane rLPS and polymyxin B.

### Biological activity of polymyxin against *E. coli* BL21

To confirm the efficacy of polymyxin against our test strain of *E. coli* BL21, we carried out susceptibility assays using the agar diffusion method.^19^ The results were obtained from three technical and three biological repeats and the minimal inhibitory concentration (MIC) was calculated, using the free MIC web calculator at http://www.agardiffusion.com, to be 291 μg ml^−1^ using the linear zone size model. The average regression coefficient for the linear d-model was *R*^2^ = 0.974, while for the d^2^ model *R*^2^ = 0.964. This closer fit to the linear model reflects some loss of polymyxin during diffusion within the agar, either through degradation or through interactions with the agarose matrix.^19^

Polymyxin B is an anti-Gram-negative peptide antimicrobial with a complex mechanism of action that involves disruption and crossing both the bacterial OM and IM. OM translocation is conditional on the availability of LPS and specific modifications, such as pEtN-ation, reduce the ability of polymyxin B to engage bacterial targets. Dye release studies in this study show that the presence of LPS in lipid membranes significantly enhances the ability of polymyxin B to destabilise and permeabilise bilayer membranes without the formation of stable non-bilayer products. While polymyxin B binds to zwitterionic PC membranes, causing a reduction in the lipid orientational order, membrane leakage only occurs at high polymyxin B to lipid ratios, most likely through charge repulsion-induced local membrane curvature. This model aligns with the reported role of membrane charge as a destabilising factor in the membrane response to polymyxin B, with better chain packing countering this effect.^26^

The presence of LPS significantly enhances the phospholipid motional freedom following the addition of polymyxin B. In contrast to the pure lipid case, a different mechanism comes to the fore, in which pyrophosphate recognition by polymyxin B leads to the assembly of lytic binary complexes that more efficiently disrupt the LPS-containing membranes. This model is consistent with reported bacterial adaptations in pEtN-derivatised LPS, which reduce bacterial susceptibility to polymyxins.^12,13^ The OM translocation of polymyxin B relies on hijacking natural LPS pyrophosphate groups, where the polycationic peptide challenges membrane integrity through competition for the divalent cation binding sites which are responsible for maintaining OM integrity. Capping LPS mono- and pyrophosphates in polymyxin resistant strains results in a reduction of the LPS negative charge, as well as restricting the access to pyrophosphates as docking motifs for polymyxin B.

## Conclusions

As last resort antimicrobials such as polymyxin B are re-entering the battle against bacterial resistance to antibiotics, novel molecular targets and antimicrobial mechanisms play an increasingly important role. In this work, we explore the role of bacterial rLPS as a molecular receptor for polymyxin B and used solid state NMR to show the specific pyrophosphate-mediated molecular interaction mechanism. We contrast and quantify non-specific membrane disruption by polymyxin B to a pyrophosphate-mediated mechanism relying on the formation of LPS/polymyxin B membrane complexes. Biologically active polymyxin B is significantly more lytic in the presence of LPS in the target membranes and solid state ^31^P MAS NMR relaxation revealed LPS pyrophosphates to be the specific loci of polymyxin B/LPS recognition.

## Conflicts of interest

The authors have no conflicts of interest to declare.

## Supplementary Material
